# The Derkay Scale as a Predictor of Voice Dysfunction in Recurrent Respiratory Papillomatosis: Correlations Between Acoustic and Patient-Reported Outcomes

**DOI:** 10.3390/jcm14197093

**Published:** 2025-10-08

**Authors:** Beata Miaśkiewicz, Elżbieta Gos, Aleksandra Panasiewicz, Paulina Krasnodębska, Dominika Oziębło, Monika Ołdak, Agata Szkiełkowska

**Affiliations:** 1 Audiology and Phoniatric Clinic, World Hearing Centre of the Institute of Physiology and Pathology of Hearing, Kajetany, 05-830 Warsaw, Poland; a.panasiewicz@ifps.org.pl (A.P.); p.krasnodebska@ifps.org.pl (P.K.); a.szkielkowska@ifps.org.pl (A.S.); 2 Department of Teleaudiology and Screening, World Hearing Centre of the Institute of Physiology and Pathology of Hearing, Kajetany, 05-830 Warsaws, Poland; e.gos@ifps.org.pl; 3 Department of Genetics, World Hearing Centre of the Institute of Physiology and Pathology of Hearing, Kajetany, 05-830 Warsaw, Poland; d.ozieblo@ifps.org.pl (D.O.); m.oldak@ifps.org.pl (M.O.)

**Keywords:** recurrent respiratory papillomatosis, dysphonia, voice parameters, voice handicap index, quality of life

## Abstract

**Objectives:** The aim of the study was to gauge the clinical usefulness of the Derkay scale in assessing the severity of voice dysfunction in patients with recurrent respiratory papillomatosis (RRP). **Material and Methods:** The study included 29 patients (8 women and 21 men) with a mean age of 40.2 years. To subjectively assess each patient’s voice, the Polish version of the Voice Handicap Index questionnaire was used. Acoustic parameters were calculated using the Multidimensional Voice Program, which included mean fundamental frequency (F0), frequency changes (% Jitter), amplitude changes (% Shimmer), noise-to-harmonic ratios (NHRs), and the soft phonation Index (SPI). The stage of RRP was assessed using the Derkay scale, together with the anatomical location of the lesion (from laryngeal endoscopy) and the impact RRP had on the general condition of the patient. **Results:** In women, Derkay clinical and total scores showed significant, positive, and strong correlations with almost all VHI-30 subscales (rho = 0.73–0.76). In men, the correlations were weaker (rho = 0.38–0.55) but were strong between the Derkay total score and F0 and total score and Jitter (rho = 0.63–0.65). Patients with human papilloma virus HPV-6 had significantly higher soft phonation index values (M = 11.97) compared to patients with HPV-11 (M = 6.91, U = 34.0; *p* = 0.019). **Conclusions:** The Derkay classification system correlates well with objective acoustic frequency measures and patient-reported voice outcomes. The system may be helpful in identifying patients at increased risk of voice dysfunction. It could be used to guide decisions about voice assessment and rehabilitation.

## 1. Introduction

Recurrent respiratory papillomatosis (RRP) is a rare disease that may cause voice disorders of varying severity and affect airway patency. It is caused by infection with the human papilloma virus (HPV). Among the HPVs responsible for the development of RRP, HPV-6 and 11, found in over 90% of RRP cases, belong to the group of low-oncogenic viruses, although type 11 is believed to be associated with a more aggressive clinical course of the disease. The less common types 16, 18, 31, and 33 have increased oncogenic risk [1,2].

RRP is classified as either the childhood form, with its onset in early childhood (Jo-RRP), or the form that develops in adulthood (Ao-RRP). The cut-off age is considered to be 12 years [3]. The incidence rate in adults is 1.8/100,000 and in children is 4.3/100,000 [4].

The gender distribution of laryngeal RRP shows a significant male predominance, which may be explained by significant differences in sex hormone levels between the sexes. Furthermore, male vocal cords have more androgen receptors than female cords. Some researchers suggest that androgens may be responsible for the higher incidence of laryngeal papilloma in men [5].

The clinical course and severity of RRP are highly variable. The most common symptom of RRP is progressive hoarseness, which causes significant voice dysfunction (reported by 68–78% of adults with RRP) [6]. Due to the involvement of the upper respiratory tract, a chronic cough, recurrent respiratory tract infection, dyspnea, and acute respiratory failure may occur. However, the biggest problem in treating this disease is the very high recurrence rate, requiring frequent reoperations.

In order to monitor the severity of papillomatous lesions in the larynx, the course of the disease, its response to treatment, and predict voice outcomes, Derkay and colleagues created a grading system based on the anatomical advancement of the lesions and their impact on the patient’s general condition. Initially created for the pediatric population, it is currently the most widely used system for assessing the stage and severity of the disease in adults as well [6,7,8].

Since good communication skills play an essential role in modern society, the negative impact of RRP and its effects on daily functioning cannot be ignored. The disease has physical, functional, and emotional impacts, and explains why voice complaints are often the primary reason for surgical treatment in RRP. Objective acoustic analysis is widely used to evaluate voice quality: it measures a range of acoustic parameters that assess frequency and amplitude disturbances and the presence of additive noise. Abnormal values of these parameters reflect structural or functional changes in vocal folds that interfere with voice production [9,10,11].

The aim of this study was to evaluate the clinical utility of the Derkay staging system in assessing the severity of voice dysfunction in patients with recurrent respiratory papillomatosis (RRP), paying particular attention to gender differences. This study sought to correlate Derkay scores with objective acoustic measures and patient-reported voice outcomes.

## 2. Materials and Methods

### 2.1. Study Setting

A single-center prospective study was performed from September 2018 to December 2024 at the Institute of Physiology and Pathology of Hearing, Kajetany/Warsaw, Poland. The research protocol and the informed consent form were approved by the Ethics Committee of the Institute of Physiology and Pathology of Hearing (IFPS:KB/15/2017). The study adhered to the principles contained in the Declaration of Helsinki.

The study included patients who had been diagnosed with RRP and who met the inclusion criteria for Cidofovir laryngeal injection therapy. Subjects were enrolled after receiving information on the details of the study and providing written informed consent at the baseline visit.

The data collected refer to the patient’s condition before starting RRP treatment with Cidofovir.

### 2.2. Inclusion and Exclusion Criteria

The inclusion criteria were the following: over 18 years old, having endoscopic features of papilloma invasion in the larynx and histopathologic diagnosis of RRP (confirmed by tests as having HPV- 6 or HPV- 11), at least one recurrence of papilloma, normal laboratory findings (including blood biometry, blood chemistry, urine, and liver tests), ability to undergo general anesthesia and endoscopic procedures safely, no prior treatment with Cidofovir for RRP, ability to attend follow-up visits and comply with study procedures, and sufficient proficiency in the study language to understand instructions and complete questionnaires.

The exclusion criteria were age under 18, papilloma in the subglottic area or trachea, abnormal laboratory tests mentioned above, known allergy or hypersensitivity to Cidofovir or its components, presence of other laryngeal tumors, inability to comply with study procedures or complete questionnaires, and pregnancy or planning pregnancy.

### 2.3. Methods

The Polish version of the Voice Handicap Index questionnaire (VHI-30) was administered to evaluate the patient’s perception of their own voice [12,13]. The VHI consists of 30 items with 5 response levels, scored 0–4. These items are divided equally among 3 subscales: functional, physical, and emotional. The functional subscale gauges the ability to communicate in various settings, the physical addresses the patient’s perceptions during production of their voice, and the emotional subscale measures emotional aspects of voice production [12]. The VHI-30 total score (VHI-T) and its components—emotional (VHI-E), physical (VHI-P), and functional (VHI-F) subscale scores—were all calculated. The maximum score of the total scale is 120 points, while each subscale has a maximum of 40 points, with higher values reflecting more severe self-perceived voice problems.

An objective acoustic voice analysis was performed using the Computerized Speech Lab (CSL) 4500 external module from Kay Elemetrics Corporation (Lincoln Park, NJ, USA). All voices were recorded in a soundproof chamber with a Behringer ECM 800 microphone positioned approximately 15 cm from the mouth and, to reduce airflow effects, at an angle of 45°. Analysis of a voice sample recorded at a sample rate of 25 kHz was performed using the Multidimensional Voice Program software (MDVP 5105 version 2.7.0). Three samples of the sustained vowel “a” in modal voice were used for analysis; only the middle portion of the uttered vowel was used (min. 0.6 s), avoiding onset and offset effects [14]. The following acoustic parameters were calculated: average fundamental frequency for all extracted pitch periods (F0), short-term frequency variation that gives an evaluation of the variability of the pitch period within the analyzed voice sample (% Jitter), short-term amplitude variation that gives an evaluation of the variability of the peak-to peak amplitude within the analyzed voice sample (% Shimmer), and two noise-related parameters (noise to harmonic ratio- an average ratio of the energy of the inharmonic components to the harmonic components, NHR, and soft phonation index- measures the harmonic structure of the spectrum, SPI).

Based on flexible nasofiberolaryngoscopy and/or laryngovideostroboscopy (LVS), all patients from the study group were qualified for surgical removal under general anesthesia of papillomatous lesions from the larynx and for subsequent local injections of Cidofovir.

RRP severity was quantified with the Derkay severity staging system, which is based on the anatomical location and advancement of the lesions (an anatomic evaluation), and their impact on the patient’s general condition (a clinical evaluation) [7]. According to the Derkay system, the aerodigestive tract is divided into 25 subsites, with each given a score of 0 to 3 (0, none; 1, surface lesion; 2, raised lesion; 3, bulky lesion). The 25 scores are calculated to generate a composite anatomical score. The complementary clinical assessment takes into account the patient’s voice (a score of 0–2), the occurrence of stridor (score of 0–2), the urgency of consultation (score of 0–3), and the level of airway safety (score of 0–4). The anatomical and clinical scores were then combined. In this study, the clinical evaluation scores were made the day before surgery. The clinical Derkay assessment was made by the operating surgeon at a preoperative consultation, and the anatomical Derkay score was calculated by the same person during the surgical procedure after a thorough intraoperative assessment using an operating microscope.

### 2.4. HPV Identification

Virus DNA was isolated from the collected tissue using the commercial Maxwell FSC DNA IQ Casework Kit and the Maxwell RSC instrument following the manufacturer’s protocol (Promega, Walldorf, Germany). Subsequently, virus genotyping was conducted using real-time PCR with a commercial probe set for two low-risk HPV genotypes (6 and 11) and 14 high-risk HPV genotypes (16, 18, 31, 33, 35, 39, 45, 51, 52, 56, 58, 59, 66, and 68). The reactions were carried out according to the standard manufacturer’s protocols (Sacace Biotechnology, Como, Italy) on the Applied Biosystems 7500 Real-Time PCR System (Life Technologies, Waltham, MA, USA).

### 2.5. Statistical Analysis

Categorical variables were summarized using percentage distributions, while continuous variables were described with appropriate descriptive statistics (mean, standard deviation, range). Relationships between Derkay scores (clinical, anatomical, and total), perceived voice handicap (measured with VHI-30), and voice acoustic parameters (assessed with MDVP) were evaluated using Spearman’s rank correlation coefficients. The Mann–Whitney test was applied to compare voice-related outcomes between women and men. A *p*-value < 0.05 was considered statistically significant. All analyses were conducted using IBM SPSS Statistics, version 24.

### 2.6. Participants

The study group comprised 29 patients (8 women and 21 men). They were aged between 20 and 80 years; the mean age was 40.2 years (SD = 11.5).

**Figure 1:**
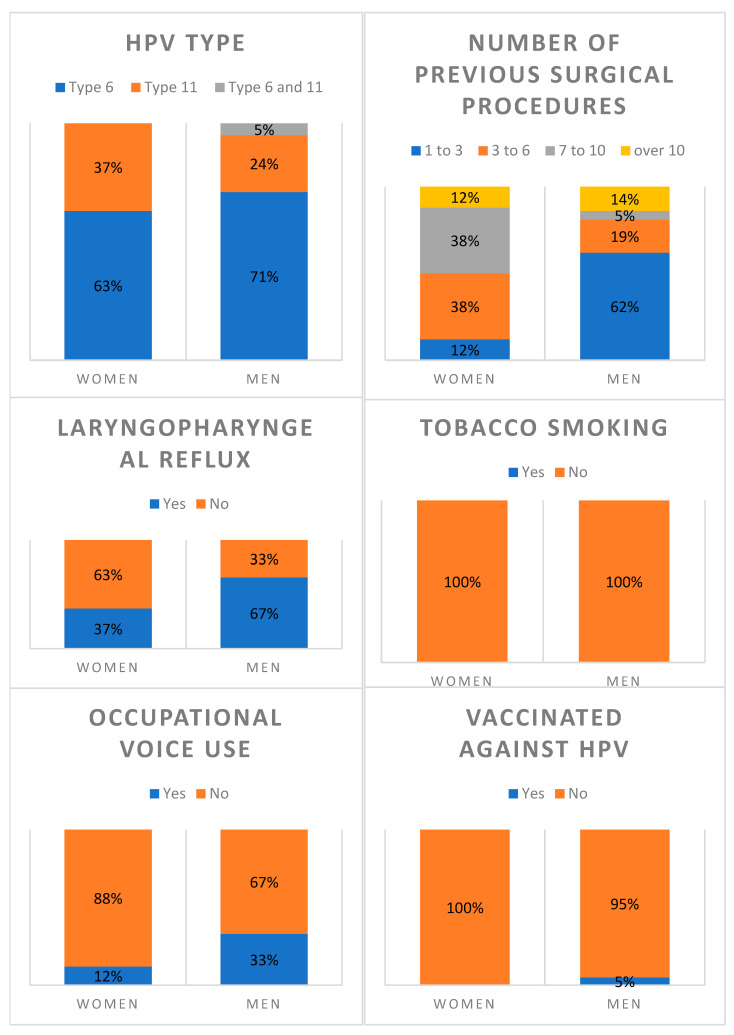
Clinical characteristics of the patients (females on left; males on right).

Table 1 and the corresponding Figure 1 summarize the clinical characteristics of the patients. The majority were infected with HPV- 6 (69%), followed by HPV- 11 (28%), with only one case of co-infection. None of the patients reported tobacco use. Occupational voice use was present in 28% of the group and laryngopharyngeal reflux was found in more than half the patients (59%). Almost all patients (97%) were not vaccinated against HPV. Regarding past surgical procedures, nearly half the patients (48%) had received between one and three surgical treatments prior to the proposed Cidofovir injection therapy, while smaller proportions had received a higher number. None of the patients had co-morbidities.

Figure 1 shows the differences in clinical features between women and men. Men reported more occupational voice use (33% vs. 12%) and laryngopharyngeal reflux (67% vs. 37%) than women. In terms of surgical history, men were more likely to have had just 1–3 previous surgeries than women (62% vs. 12%), whereas those patients who had undergone more than 3 procedures were more often women.

## 3. Results

### 3.1. Relationship Between Derkay Scores and VHI-30

The correlations between Derkay scores (clinical, anatomical, and total) and the Voice Handicap Index (VHI-30) scores (functional, emotional, physical, and total) are presented in Table 2. Analysis was performed for the entire patient group (*n* = 29), as well as separately for women (*n* = 8) and men (*n* = 21). Statistically significant positive correlations were observed, indicating that higher disease severity, as measured by the Derkay scale, was associated with greater voice-related handicap, particularly in the functional and physical domains.

In all patients, significant, positive and moderate correlations were observed between all Derkay subscores (clinical, anatomical, and total) and the VHI-30 total score as well as its subscales (rho = 0.48–0.59). This indicates that greater disease severity, both anatomically and symptomatically, is associated with worse perceived voice handicap. However, these relationships were more pronounced in the functional and physical domains, while slightly weaker correlations were noted in the emotional domain.

In women, the clinical and total Derkay scores showed significant, positive, and strong correlations with most of the VHI-30 subscales (rho = 0.73–0.76). Although the anatomical score also showed moderately high correlations (rho = 0.61–0.66), these did not reach statistical significance due to the small sample size. This suggests that in women, subjective voice handicap is more sensitive to disease severity than to anatomical features, especially clinical symptoms.

In men, the correlations were weaker compared to women, ranging from rho 0.38 to 0.55. Significant associations were found for the Physical and Functional VHI-30 scales, but not the Emotional, where correlations were weaker and not statistically significant. This suggests that men’s perceived voice handicap is less closely related to disease severity than women’s, particularly in the emotional domain.

### 3.2. Relationship Between Derkay Scores and MDVP

Table 3 presents the correlations between Derkay scores (clinical, anatomical, and total) and acoustic voice parameters measured by the Multidimensional Voice Program (MDVP).

In the total sample, moderate and statistically significant positive correlations were found between Derkay scores and several acoustic measures, particularly jitter (rho up to 0.56) and, to a lesser extent, shimmer and F0. These results suggest that greater disease severity is associated with increased frequency and amplitude perturbations. No significant associations were observed with NHR or SPI.

Sex-specific analysis revealed stronger correlations in men, especially between anatomic and total Derkay scores and both F0 and Jitter (rho = 0.63–0.65). Correlations in women were weaker and statistically nonsignificant, probably because of the smaller sample size. Interestingly, the direction of correlations between NHR and SPI differed between women and men, suggesting sex-specific differences in how disease severity affects noise components and phonatory control.

### 3.3. Comparison of Voice-Related Outcomes Between Women and Men

To further investigate the observed differences in correlations between clinical variables and voice measures by sex, additional analyses were conducted to compare the levels of Derkay scores, VHI-30 scores, and MDVP parameters between women and men (Table 4).

The results show that men had slightly higher mean scores than women in all Derkay dimensions (clinical, anatomical and total), indicating a trend towards greater disease severity, although these differences were not statistically significant.

Similarly, in the VHI-30 domains, men reported slightly higher functional and physical disability scores, while women had slightly higher emotional scores, but no statistically significant differences were found between the sexes (*p* > 0.05 for all comparisons).

A statistically significant difference was observed for fundamental frequency (F0), with women having a higher mean F0 (230.7 Hz) compared to men (158.6 Hz; *p* = 0.004), consistent with typical gender differences in vocal pitch. But for the remaining MDVP parameters (Jitter, Shimmer, NHR, SPI), the results were not statistically significant.

### 3.4. Voice-Related Outcomes in Relation to Clinical Features

The potential influence of clinical features on voice-related outcomes was analyzed. Some differences were observed depending on the HPV type. Patients infected with HPV type 6 showed slightly higher Derkay anatomical and total scores compared to those with HPV type 11; however, these differences did not reach statistical significance. Likewise, the VHI-30 Functional and Physical subscale scores were somewhat higher in patients with HPV type 6, but again without statistically significant differences. The only statistically significant difference was found in the acoustic parameter related to soft phonation (Soft Phonation Index, SPI). Patients with HPV type 6 demonstrated significantly higher SPI values (M = 11.97; SD = 5.76) compared to those with HPV type 11 (M = 6.91; SD = 2.79), U = 34.0; *p* = 0.019.

No statistically significant associations were found between the presence of laryngopharyngeal reflux and Derkay scores, VHI-30 scores, MDVP parameters, or the number of surgical procedures. Similarly, occupational voice use was not associated with any of these outcomes.

## 4. Discussion

The frequent recurrent nature of RRP and the therapies used against it have a large impact on the overall quality of patient life. Progressive hoarseness, found in 68–78% of adults with RRP, is often a major factor in decisions to proceed with surgical treatment [15]. The methods of disease grading mentioned in the literature, mainly in relation to Jo-RRP, help doctors to make individualized treatment plans, including the selection of patients for surgical treatment, planning of surgery or further follow-up [7,8,16].

The widely used Derkay staging system enables a functional assessment of disease severity as well as an anatomical assessment of where it is occurring. The Derkay system is successfully used in both children and adults, which is confirmed by other researchers, who indicate a high level of surgeon-to-surgeon reliability [17,18]. Nevertheless, Hock et al., in order to reduce inter-rater variability, implemented the calculation of the Derkay score only by one reviewer, similar to what was performed in our study [17]. Although the Derkay scale is the most commonly used method in the literature, also in adults, its main limitation is the need for complete exposure of the lesion, which is usually only possible intraoperatively, during the surgical procedure [16].

Our results demonstrate a significant and strong positive correlation between the Derkay staging system and patients’ self-assessed voice disability, as measured using the VHI-30 questionnaire (Table 3). These results are in line with the findings of Kupfer and colleagues and further support the use of VHI scores as a reliable indicator of RRP severity [6].

Despite the small number of women and the uneven gender distribution, a prognostic observation in our study is that in women, most correlations were stronger than in men, notably for the statistically significant Derkay clinical and total subcores, with the exception of the correlation with the emotional subscale of the VHI and the Derkay total score. At the same time, in men, these correlations were generally weaker and for the VHI emotional subscale did not reach statistical significance. These results indicate that the functional and physical aspects assessed by both the VHI and the Derkay scale are more important for women than for men and suggest a less significant influence of voice dysfunction on the emotional state in men. However, due to the small number of women in the study group, these results are preliminary and should be treated with caution.

The research by Nieuwenhuizen and colleagues on a group of 34 patients with RRP did not show any relationship between VHI and gender, number of procedures, or location of lesions. However, they noted that voice dysfunction was significantly predicted by a shorter time since the last procedure and a more passive coping style [15].

The presence of exophytic papillomatous lesions within the laryngeal structures, especially on the vocal folds, together with often multiple phonosurgical treatments, tends to affect the condition of the multilayer structure of the vocal folds, and thus affect the pitch of the voice. Acoustic parameters such as Jitter and Shimmer reflect cycle-by-cycle frequency and amplitude variations, and these are known to correlate with perceptual ratings of hoarseness [19]. Even very small exophytic lesions on vocal folds can affect the symmetry of vibrations and perturb frequencies [20]. Other researchers indicate a significant increase in the values of parameters defining the amplitude (Shimmer) and frequency (Jitter) of laryngeal tone [10] as well as NHR in patients with laryngeal RRP [11]. Our results confirm these observations and show that greater disease severity is associated with increased frequency and amplitude perturbation, as indicated by moderate and statistically significant positive correlations between Derkay scores and jitter, shimmer, and F0. Lehto and colleagues compared RRP patients and healthy controls and observed significantly increased F0 values and slightly increased jitters in their papillomatous group, although NHR values were similar in both groups [21]. In line with these findings, in our study, we did not observe a significant association between Derkay score and NHR or SPI.

Regarding gender, our results show stronger correlations in men than in women, especially between anatomical and total Derkay scores and both F0 and Jitter. This may suggest a greater influence of the extent of anatomic involvement on frequency-related acoustic parameters in men than in women.

Again, in relation to gender, we also found negative correlations in NHR in women and SPI in men, which is promising but requires further investigation. As Yumoto et al. noted, the H/N ratio shows significant correlation with the psychophysical measurement of the degree of hoarseness [22]. Incomplete glottal closure will cause turbulence and irregularity in sound waves, measured as high-frequency noise [21]. It can be speculated that the negative correlation with NHR in women results from anatomical conditions, i.e., the smaller size of the larynx and thus the smaller surface of the glottis.

High values of SPI are stated to correlate with incomplete vocal fold adduction and can be used as an indicator of breathiness [23]. However, increases in SPI are also noted for pressed phonation [24]. The negative correlation observed in men between the Derkay score and SPI values may be explained by the fact that exophytic papillomatous changes in the vocal folds reduce the area of glottal gap. In turn, the positive correlation for SPI observed in women may result from the coexisting increased component of supraglottic hyperfunction, which is more common in women and may refer to the higher emotional score in VHI.

When comparing the data obtained for both sexes, we did not obtain statistically significant differences except for F0, the value of which was significantly higher in women than in men, which, however, results from anatomical and physiological differences between the sexes.

In terms of patient-reported voice outcomes, the impact of emotion on quality of life is more noticeable in women than in men, who tend to score higher on functional and physical disability scores.

However, due to the uneven gender distribution and the small number of women, the above results regarding gender differences should be treated with caution.

There was no correlation between voice-related outcome (Derkay score, acoustic parameters, and VHI) and clinical features, except for the SPI, which was significantly higher in HPV-6 infected patients, who also had slightly higher Derkay anatomical and total scores and the VHI-30 Functional and Physical subscale scores compared to HPV-11 patients.

The main limitation of our study is the small size of the group, especially women, which means that the observed trends in intersex differences in the analyzed correlations require further research on a larger group of patients. Because the present paper is based on a prospective study that is still ongoing, we expect a larger cohort for further research.

## 5. Conclusions

Our findings suggest that the Derkay staging system meaningfully correlates with objective acoustic frequency-related measures and patient-reported voice outcomes. This highlights its potential utility not only in monitoring disease burden, but also in evaluating the impact of recurrent respiratory papillomatosis (RRP) on vocal function. In clinical practice, the Derkay score may help identify patients at higher risk of voice dysfunction, guiding decisions regarding voice assessment and early rehabilitation. Despite the uneven gender distribution, which affects the more exploratory and prognostic nature of the results, in men, the anatomical extent of disease appears to be more strongly associated with objective voice abnormalities, whereas in women, the functional impact reflected in the Derkay clinical score may better predict perceived voice handicap.

Although men showed a trend toward higher Derkay scores in all dimensions, these sex-related differences warrant further investigation to tailor management strategies.

## Figures and Tables

**Table 1:** Clinical characteristics of patients.

		*n*	*%*
HPV type	6	20	69%
	11	8	28%
	6 and 11	1	3%
Tobacco smoking	Yes	0	0%
	No	29	100%
Occupational voice use	Yes	8	28%
	No	21	72%
Laryngopharyngeal reflux	Yes	17	59%
	No	12	41%
Vaccinated against HPV	Yes	1	3%
	No	28	97%
Number of treatments before proposed injection	1–3	14	48%
	4–6	7	24%
	7–10	4	14%
	Over 10	4	14%
Time passed since the last treatment	Up to 6 months	6	21%
	6–12 months	15	52%
	Over 12 months	8	27%

**Table 2:** Correlations (rho) between Derkay scores (clinical, anatomical, and total) and VHI-30 scores.

Derkay Score		VHI-30			
		Functional	Emotional	Physical	Total
All patients (*n* = 29)	Clinical	0.55 *	0.49 **	0.58 **	0.54 **
	Anatomical	0.57 **	0.48 **	0.58 **	0.54 **
	Total	0.58 **	0.48 **	0.59 **	0.55 **
Women (*n* = 8)	Clinical	0.76 *	0.76 *	0.76 *	0.76 *
	Anatomical	0.66	0.61	0.66	0.66
	Total	0.73 *	0.68	0.73 *	0.73 *
Men (*n* = 21)	Clinical	0.44 *	0.38	0.50 *	0.43 *
	Anatomical	0.50 *	0.41	0.55 *	0.49 *
	Total	0.50 *	0.41	0.55 *	0.49 *

** *p* < 0.01, * *p* < 0.05.

**Table 3:** Correlations between Derkay scores (clinical, anatomical, and total) and MDVP scores.

		MDVP				
		F0	Jitt	Shim	NHR	SPI
All patients (*n* = 29)	Clinical	0.27	0.48 **	0.39 *	0.21	−0.14
	Anatomical	0.44 *	0.55 **	0.27	0.21	−0.13
	Total	0.44 *	0.56 **	0.27	0.22	−0.11
Women (*n* = 8)	Clinical	0.50	0.50	0.50	−0.13	0.38
	Anatomical	0.34	0.39	−0.05	−0.48	0.46
	Total	0.40	0.44	0.01	−0.43	0.42
Men (*n* = 21)	Clinical	0.53 *	0.42	0.29	0.26	−0.38
	Anatomical	0.65 **	0.63 **	0.34	0.38	−0.28
	Total	0.65 **	0.63 **	0.34	0.37	−0.27

** *p* < 0.01, * *p* < 0.05.

**Table 4:** Comparison of Derkay scores, VHI-30 scores, and MDVP parameters between female and male patients.

		Women (*n* = 8)		Men (*n* = 21)		Test Statistic	*p*
		M	SD	M	SD		
Derkay	Clinical	0.75	0.46	0.95	0.38	68.0	0.234
	Anatomical	6.88	5.11	8.38	5.77	69.5	0.478
	Total	7.63	5.37	9.33	6.04	68.0	0.434
VHI-30	Functional	12.75	10.71	14.38	10.33	77.0	0.732
	Emotional	14.63	10.68	13.38	10.84	80.5	0.864
	Physical	16.63	12.07	18.57	9.34	76.0	0.696
	Total	44.00	32.70	46.19	29.89	79.5	0.826
MDVP	F0	230.71	60.78	158.60	55.76	25.0	0.004
	Jitt	2.15	1.71	4.23	4.32	52.0	0.118
	Shim	6.15	2.17	9.28	6.79	61.0	0.262
	NHR	0.15	0.03	0.29	0.22	48.0	0.079
	SPI	8.35	2.90	11.45	6.01	55.0	0.157

M—mean; SD—standard deviation.

## Data Availability

The original contributions presented in this study are included in the article. Further inquiries can be directed to the corresponding author(s).

## Abbreviations

The following abbreviations are used in this manuscript:

RRP	recurrent respiratory papillomatosis
F0	mean fundamental frequency
NHR	noise to harmonic ratio
SPI	soft phonation index
HPV	human papilloma virus
Jo-RRP	recurrent respiratory papillomatosis with its onset in early childhood
Ao-RRP	recurrent respiratory papillomatosis which develops in adulthood
VHI-30	voice handicap index
VHI-T	voice handicap index total score
VHI-E	voice handicap index emotional score
VHI-P	voice handicap index physical score
VHI-F	voice handicap index functional
CSL	computerized speech Lab
MDVP	multidimensional voice program
LVS	laryngovideostroboscopy
